# *In vivo* total or partial hepatectomy followed by *ex vivo* liver resection and autotransplantation for malignant tumors: a single center experience

**DOI:** 10.3389/fonc.2023.1214451

**Published:** 2023-06-23

**Authors:** Shaoyan Xu, Chenlu Hu, Zedong Jiang, Guogang Li, Bo Zhou, Zhenzhen Gao, Weilin Wang, Sheng Yan

**Affiliations:** ^1^ Division of Hepatobiliary and Pancreatic Surgery, Department of Surgery, Second Affiliated Hospital, School of Medicine, Zhejiang University, Hangzhou, Zhejiang, China; ^2^ Key Laboratory of Precision Diagnosis and Treatment for Hepatobiliary and Pancreatic Tumor of Zhejiang Province, Second Affiliated Hospital of Zhejiang University School of Medicine, Hangzhou, Zhejiang, China; ^3^ Department of Nursing, Second Affiliated Hospital, School of Medicine, Zhejiang University, Hangzhou, Zhejiang, China

**Keywords:** *in vivo* total hepatectomy, *in vivo* partial hepatectomy, hepatic metastases, autotransplantation, biliary tract cancer

## Abstract

**Background:**

*Ex vivo* liver resection and autotransplantation (ELRAT) may provide an opportunity for R0 resection of conventionally unresectable hepatobiliary cancers and hepatic metastases. To date, few studies of the surgery for malignant tumors have been conducted and there are no known reports of *in vivo* partial hepatectomy followed by ELRAT (IPH-ELRAT) for malignant tumors.

**Methods:**

Between December 2021 and November 2022, ten patients with malignant hepatobiliary primary cancers or hepatic metastases underwent ELRAT at our institution. We shared the surgical skills and postoperative prognoses of these patients were assessed.

**Results:**

The types of tumors were biliary tract cancer (BTC, n=8), hepatic metastasis of colonic carcinoma (n=1), and hepatic metastasis of small-bowel stromal tumor (n=1). Five patients underwent *in vivo* total hepatectomy followed by *ex vivo* liver resection and autotransplantation (ITH-ELRAT), The other five received *in vivo* partial hepatectomy followed by *ex vivo* liver resection and autotransplantation (IPH-ELRAT). Four patients underwent inferior vena cava replacement using artificial blood vessels. The survival rate of all ten patients one month after surgery was 100%. Nine patients (90%) are currently alive, with a median follow-up of 8.5 months (range 6–16.5 months). To date, seven of the nine surviving patients have had no cancer recurrence, including six with BTC.

**Conclusions:**

We report the world first five cases that received IPH-ELRAT for malignancies. We also demonstrated relatively favorable outcomes in patients who underwent ELRAT. ELRAT may be a recommendable surgical option for selected patients with conventionally unresectable hepatobiliary malignant tumors.

## Introduction

Hepatobiliary primary cancers include hepatocellular carcinoma (HCC) and biliary tract cancer (BTC). HCC is the sixth most common malignant tumor worldwide and ranks as the fourth leading cause of cancer-related death (1). The incidence of BTC is relatively low, which refers to a group of cancers, that include cholangiocarcinoma (cancers arising in the intrahepatic, perihilar, or distal biliary tree) and gallbladder carcinoma. The incidence of cholangiocarcinoma is 0.35–2 cases per 100,000 each year in high-income countries. While, the incidence in endemic regions of China is up to 40x higher. Meanwhile, the incidence of intrahepatic cholangiocarcinoma is increasing in high-income countries (2). Curative surgical resection is the most recommended treatment for patients with early-stage BTC (2, 3). However, most patients with these malignant tumors are diagnosed at an advanced stage when they have already missed the opportunity for traditional curative resection (2, 4). The liver is a common site for metastases (5) and it is recommended that metastatic liver tumors, such as those from colorectal carcinoma (CRC) and gastrointestinal stromal tumor (GIST), should be treated using resection in conjunction with systemic therapy (5, 6).

Conversion therapy aims to downstage tumors so that patients with initially unresectable or borderline resectable malignancies can undergo radical resection. This method has been used to treat various solid tumors, such as colorectal, pancreatic, gastric, and hepatobiliary cancer (7–9). Since 2017, combination treatment with the anti-angiogenesis targeting agent of lenvatinib and anti-programmed cell death protein 1 (PD-1) antibodies, has achieved high therapeutic response rates for HCC and BTC (10). A high overall response rate (ORR) (42.1%) was obtained in a phase II study of first-line treatment for initially unresectable BTC using lenvatinib and PD-1 inhibitors (11). In another phase II study of patients receiving toripalimab combined with lenvatinib and chemotherapy of GEMOX, a higher ORR (80%) was achieved (12). Although the combination therapy has not yet become first-line therapy for unresectable HCC and BTC patients in China, these high tumor treatment response rates of the therapy had converted some unresectable hepatobiliary cancers into resectable in some clinical practice (7, 11). Conversion therapy has also been used to treat liver metastases from colonic carcinomas (13) and metastases from gastrointestinal stromal tumors (14).

While systematic treatment is effective in some patients, however, tumors that invade important blood vessels such as the inferior vena cava, hepatic arteries, and veins, cannot be resected using traditional partial hepatectomy. Improvements in liver resection and liver transplantation technologies have allowed surgeons to explore *ex vivo* liver resection and autotransplantation (ELRAT) (15). This surgery was first performed by Pichlmayr et al. in 1988 (16). Its primary purpose is to achieve radical (R0) resection in demanding cases (17, 18). ELRAT involves *in vivo* hepatectomy and extracorporeal liver resection, followed by autologous transplantation of the remnant hepatic parenchyma. Surgery involving *in vivo* total hepatectomy followed by *ex vivo* liver resection and autotransplantation (ITH-ELRAT) for malignant tumors has been used for more than 100 patients (15, 19). While, surgery involving *in vivo* partial hepatectomy followed by *ex vivo* liver resection and autotransplantation (IPH-ELRAT) has not yet been used for patients with malignant tumors. The use of this procedure, which was called auxiliary partial autologous liver transplantation, has only been reported in one patient with hepatic alveolar echinococcosis (20). ELRAT has been performed for patients with various types of liver tumors, including malignancies (e.g., HCC, BTC, CRC metastasis, and GIST metastasis) and benign lesions including hepatic alveolar echinococcosis, focal nodular hyperplasia, and hemangioma (19, 21, 22). Of these, alveolar echinococcosis is the most frequently reported disease (23). There have been few reports on the use of ELRAT for malignant cases due to the extremely challenging nature and questionable efficacy of the surgical procedure (19). To date, there are only two studies that reported at least 10 cases of ELRAT for malignancies (15, 24). In the present study, we report ten patients with malignant tumors who underwent ELRAT and analyzed systemic therapy, surgical skills, and postoperative prognoses were assessed. Five of the cases received IPH-ELRAT and were the first to ever undergo this treatment for malignant tumors.

## Materials and methods

### Participants

Ten patients who underwent ELRAT at the Second Affiliated Hospital, School of Medicine at Zhejiang University between December 2021 and November 2022 were retrospectively analyzed. The study was approved by the Medical Science Ethics Committee of our hospital. Six patients receiving preoperative systemic therapy were fully informed and signed informed consent, including four BTC patients receiving combination therapy with lenvatinib, PD-1 inhibitor, and chemotherapy. All ten cases were discussed by a multidisciplinary tumor board. Each patient underwent a thorough evaluation and consented to a full understanding of the benefits and risks of the procedures, including prolonged hospitalization, decreased quality of life, severe complications, and even death.

### Statistical analyses

The clinical data were collected and displayed using descriptive statistics. All analyses were performed using SPSS version 26.0.

## Results

Detailed information on patient demographics, tumor location, vascular involvement, systemic therapy, operative details, and outcomes are shown in **Tables 1**, **2**. A summary of the findings is shown in **Table 3**.

**Table 1:** Patient demographics, types of tumors, location, vascular involvement, systemic therapy and follow-up.

Cases	Age/sex	Tumor types	Location	Vascular involvement	Preoperative systemic therapy, duration (months)	Follow-up(months)
1	69/F	Intrahepatic cholangiocarcinoma	I	RPV, MHV, IVC	Lenvatinib+PD-1 inhibitor+chemotherapy, 2	16.5, Alive
2	66/M	Intrahepatic cholangiocarcinoma	I, V, VI, VIII	IVC	Lenvatinib+PD-1 inhibitor+chemotherapy, 5	13, Alive
3	50/M	Metastases from colonic carcinoma	I, IV	MPV, IVC	Chemotherapy+Erbitux, 4	13, Alive
4	52/M	Hilar cholangiocarcinoma	Hilar bile duct, VI	LHA, LPV , LHV, IVC	Lenvatinib+PD-1 inhibitor+chemotherapy, 2	9, Alive
5	58/M	Metastasis from small bowel stromal tumors	I, V, VI, VII, VIII	IVC	Imatinib mesylate, 96	8, Alive
6	69/F	Hilar cholangiocarcinoma	Hilar bile duct, the lower part of the common bile duct	PV, RHA	None	2, Died
7	46/F	Gallbladder carcinoma	Gallbladder, hilar bile duct, antrum, duodenum	PV, RHV	Lenvatinib+PD-1 inhibitor+chemotherapy, 6	8.5, Alive
8	67/M	Hilar cholangiocarcinoma	Hilar bile duct	RHA	None	6, Alive
9	69/M	Hilar cholangiocarcinoma	Hilar bile duct, IVb	PV, RHV	None	6, Alive
10	67/M	Hilar cholangiocarcinoma	Hilar bile duct	PV, RHV	None	6, Alive

PV, portal vein; LPV, left portal vein; RPV, right portal vein; LHA, left hepatic artery; RHA, right hepatic artery; LHV, left hepatic vein; RHV, right hepatic vein; MHV, middle hepatic vein; IVC, inferior vena cava.

**Table 2:** Details of surgical procedure.

Cases	Removed liver segments	Reconstruction of vascular system	Vascular graft types	Operation time (mins)	Blood loss (ml)	Procedures
1	I, II, III, IV	MHV, IVC	Allogeneic iliac vein	740	400	ITH-ELRAT
2	I, V, VI, VII, VIII	MHV, IVC	Artificial blood vessel	550	500	ITH-ELRAT
3	I, II, III, IV	MHV, IVC	Artificial blood vessel	800	1600	ITH-ELRAT
4	I, II, III, IV, partial VI	IVC	Artificial blood vessel	615	1500	ITH-ELRAT
5	I, V, VI, VII, VIII	IVC	Artificial blood vessel	670	300	ITH-ELRAT
6	V, VIII	PV, RHA	None	890	300	IPH-ELRAT and Whipple
7	V, VIII	PV, RHV	None	725	1000	IPH-ELRAT and Whipple
8	V, VIII	PV, RHV	None	735	800	IPH-ELRAT
9	V, VIII, partial IVb	PV, RHV	Artificial blood vessel	650	300	IPH-ELRAT
10	V, VIII	PV, RHV	Artificial blood vessel, autogenous left great saphenous vein	815	1000	IPH-ELRAT

ITH-ELRAT, *in vivo* total hepatectomy and then *ex vivo* liver resection and autotransplantation; IPH-ELRAT, *in vivo* partial hepatectomy and then *ex vivo* liver resection and autotransplantation; PV, portal vein; LPV, left portal vein; RPV, right portal vein; LHA, left hepatic artery; RHA, right hepatic artery; LHV, left hepatic vein; RHV, right hepatic vein; MHV, middle hepatic vein; IVC, inferior vena cava.

**Table 3:** Summary of characteristics data from 10 patients receiving ELRAT.

	n (%) or median (range) In total (n=10)	ITH-ELRAT (n=5)	IPH-ELRAT (n=5)
Age (years)	66.5 (46–69)	58 (50-69)	67 (46-69)
Sex (male/female), (male %)	7/3 (70%)	4/1 (80%)	3/2 (60%)
Types of tumors			
Biliary tract cancers	8 (80%)	3 (60%)	5 (100%)
Metastasis from colonic carcinoma	1 (10%)	1 (20%)	0 (0%)
Metastasis from small bowel stromal tumor	1 (10%)	1 (20%)	0 (0%)
Preoperative adjuvant therapy	6 (60%)	5 (100%)	1 (20%)
Postoperative adjuvant therapy	9 (90%)	5 (100%)	4 (80%)
ELRAT and Whipple	2 (20%)	0 (0%)	2 (40%)
R0 resection	10 (100%)	5 (100%)	5 (100%)
Estimated blood loss (mL)	650 (300–1600)	500 (300-1600)	800 (300-1000)
HTK solution	10 (100%)	5 (100%)	5 (100%)
Duration of surgery (mins)	730 (550–890)	670 (550-800)	735 (650-890)
Length of postoperative hospital stay (days)	18 (13–30)	16 (13-21)	23 (15-30)
Follow-up (months)	8.25 (2–16.5)	13 (8-16.5)	6 (2-8.5)
Recurrence	2 (20%)	2 (40%)	0 (0%)
1 month mortality, n (%)	0 (0%)	0 (0%)	0 (0%)
6 months mortality, n (%)	1 (10%)	0 (0%)	1 (20%)

ELRAT, *ex vivo* liver resection and autotransplantation; ITH-ELRAT, *in vivo* total hepatectomy followed by *ex vivo* liver resection and autotransplantation; IPH-ELRAT, *in vivo* partial hepatectomy followed by *ex vivo* liver resection and autotransplantation.

### Patient demographics and clinical features

Ten patients (seven males and three females) had a median age of 66.5 years (range, 46–69 years). All patients were considered ineligible for conventional liver resection because of the difficult anatomical location of their tumors. The tumors were BTC (n=8, five hilar cholangiocarcinomas, two intrahepatic cholangiocarcinomas, and one gallbladder carcinoma), hepatic metastasis of colonic carcinoma (n=1), and hepatic metastasis of small-bowel stromal tumor (n=1).

### Preoperative systemic therapy

Six of the ten patients received preoperative systemic therapy, and four of the eight patients with BTC received combination therapy with lenvatinib, PD-1 inhibitor, and chemotherapy. A patient with colonic carcinoma liver metastases received chemotherapy and Erbitux and a patient with liver metastasis from a small intestinal stromal tumor received imatinib mesylate prior to ELRAT.

### Procedures and operative parameters

All patients underwent *ex vivo* hepatectomy followed by liver autotransplantation, and two (cases 6 and 7) underwent a synchronous Whipple procedure. The median duration of surgery was 730 min (range, 550–890 min) and the median estimated blood loss was 650 mL (range, 300–1600 mL). After liver resection, it was perfused with histidine-tryptophan-ketoglutarate (HTK) solution through the portal vein. Four patients (cases 2, 3, 4, and 5) underwent inferior vena cava replacement using artificial blood vessels. Case 9 underwent portal vein reconstruction using an artificial blood vessel, case 10 underwent portal vein and right hepatic artery reconstruction with an artificial blood vessel and an autogenous left great saphenous vein, respectively, and case 1 underwent reconstruction of the second hepatic hilum using an iliac vein allograft. All patients received R0 resection.

**Figure 1:**
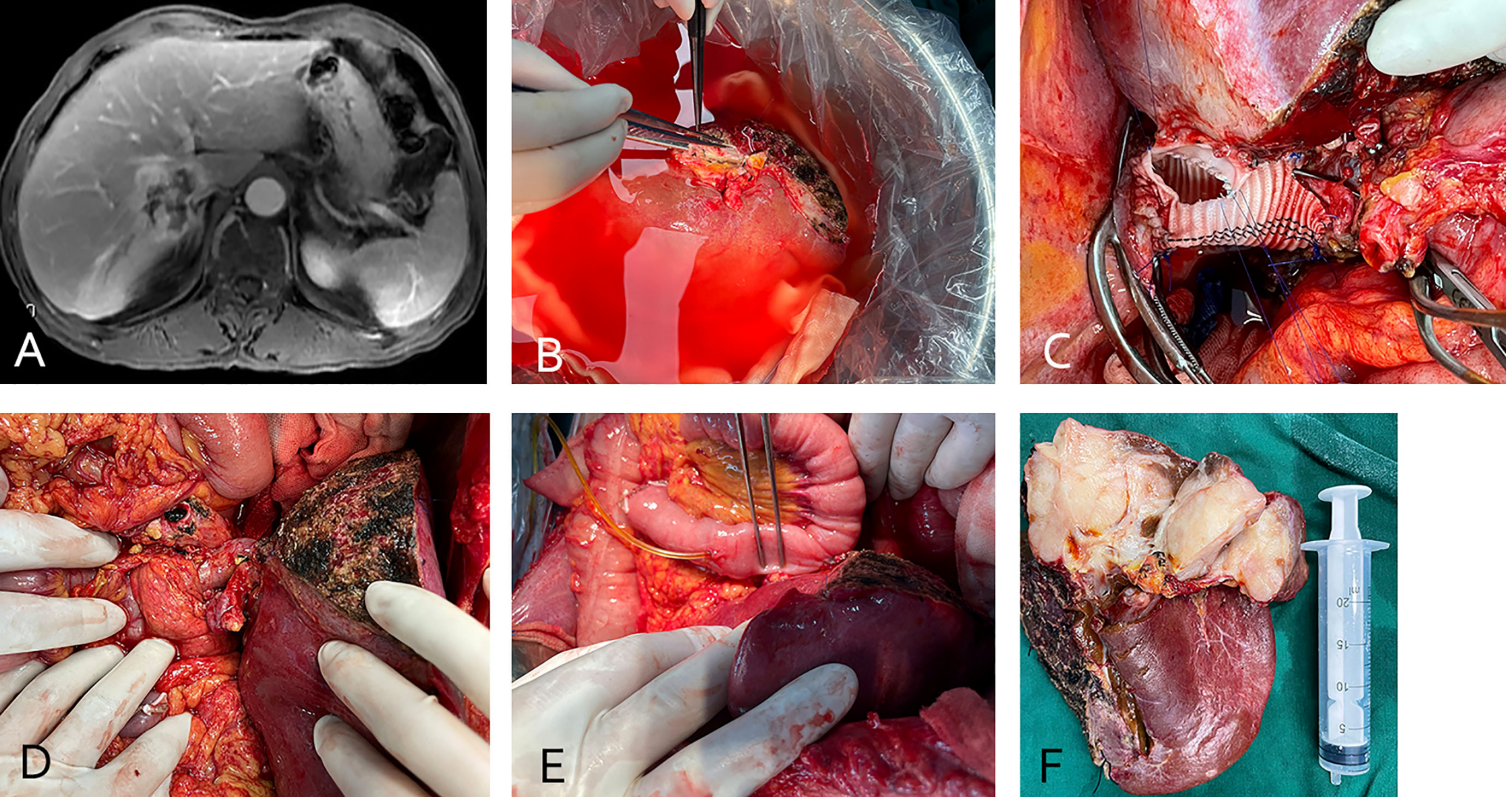
(case 2). A 66-year-old male intrahepatic cholangiocarcinoma patient with vascular involvement of the inferior vena cava **(A)**. The patient underwent *in vivo* total hepatectomy, the tumor was resected at the back table and an autograft was created (the right liver was preserved) **(B)**. Artificial blood vessel replacement of the inferior vena cava and venous anastomosis **(C)**. After anastomosis of the portal vein and hepatic artery **(D)**, bilioenteric anastomosis and external biliary drainage **(E)**. The excised specimen **(F)**.

**Figure 2:**
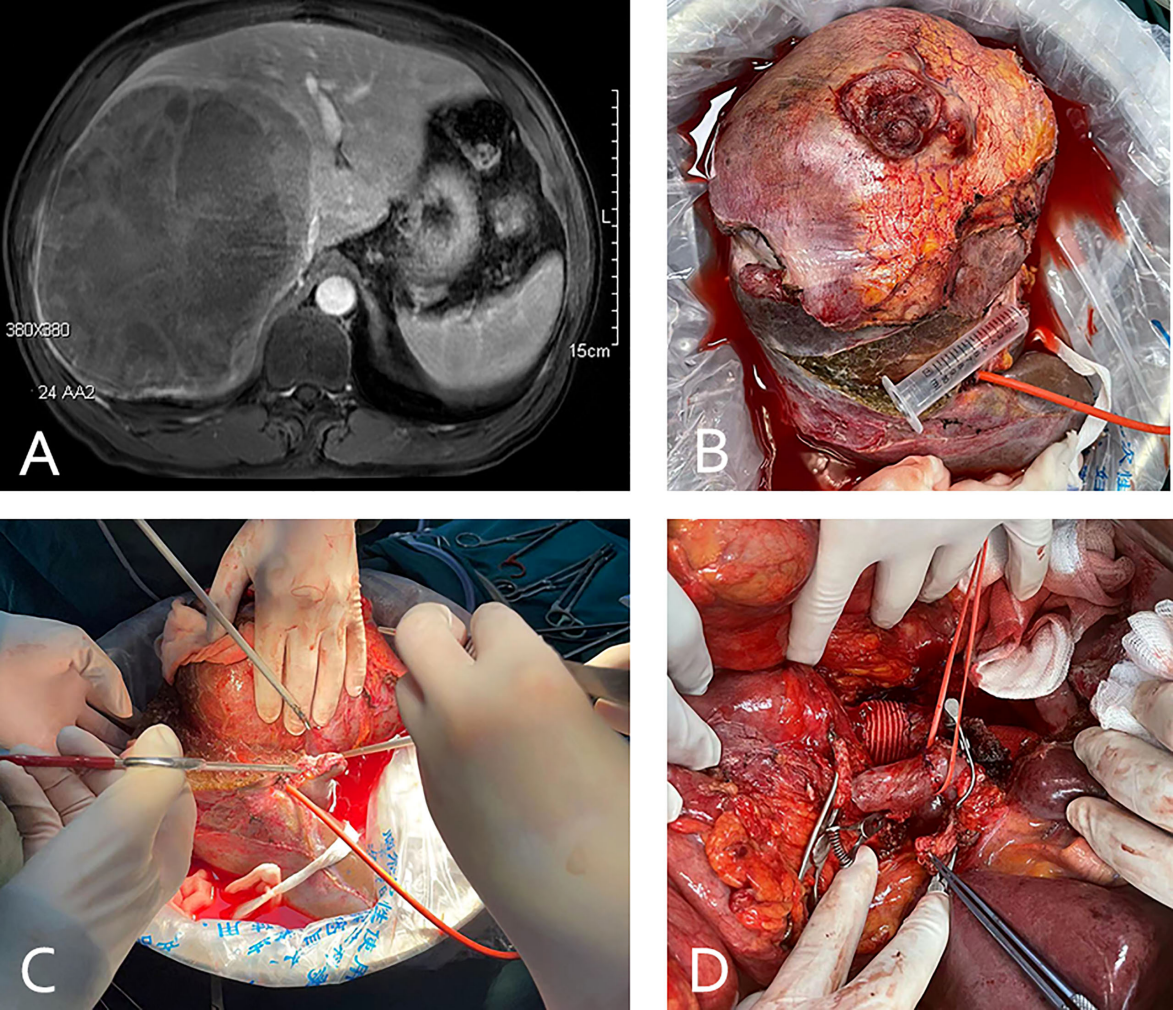
(case 5). A 58-year-old male patient with small bowel stromal tumor metastasis and vascular involvement of the inferior vena cava **(A)**. The patient underwent *in vivo* total hepatectomy, the tumor was resected at the back table and an autograft of the right liver was created **(B, C)**. Artificial blood vessel replacement of the inferior vena cava and anastomosis of the portal vein and hepatic artery were performed **(D)**.

**Figure 3:**
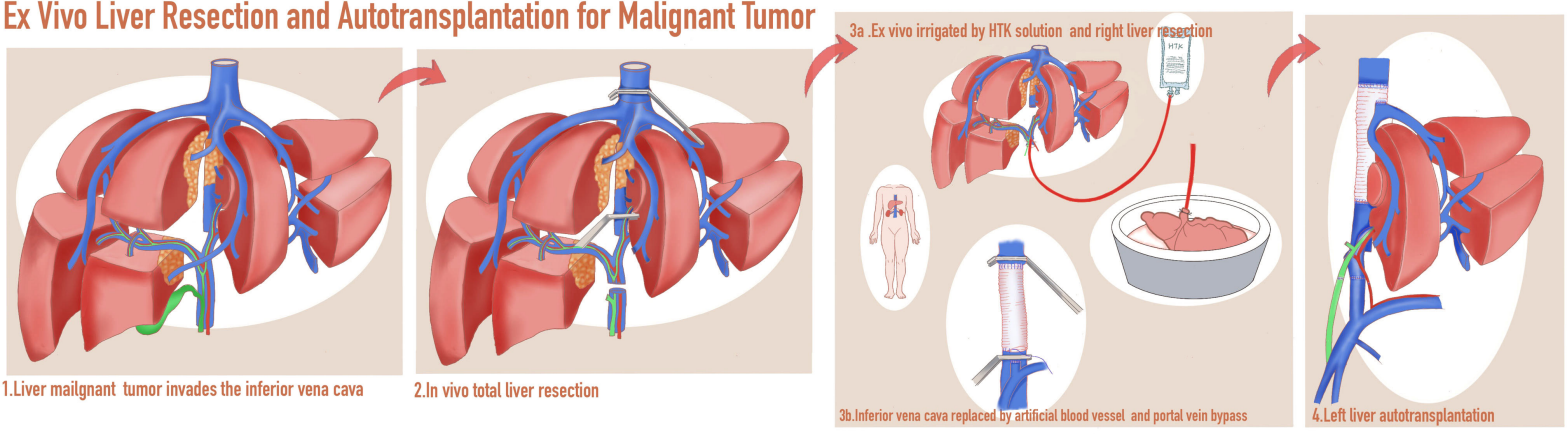
A schematic diagram of *in vivo* total hepatectomy followed by *ex vivo* liver resection and autotransplantation (ITH-ELRAT).

**Figure 4:**
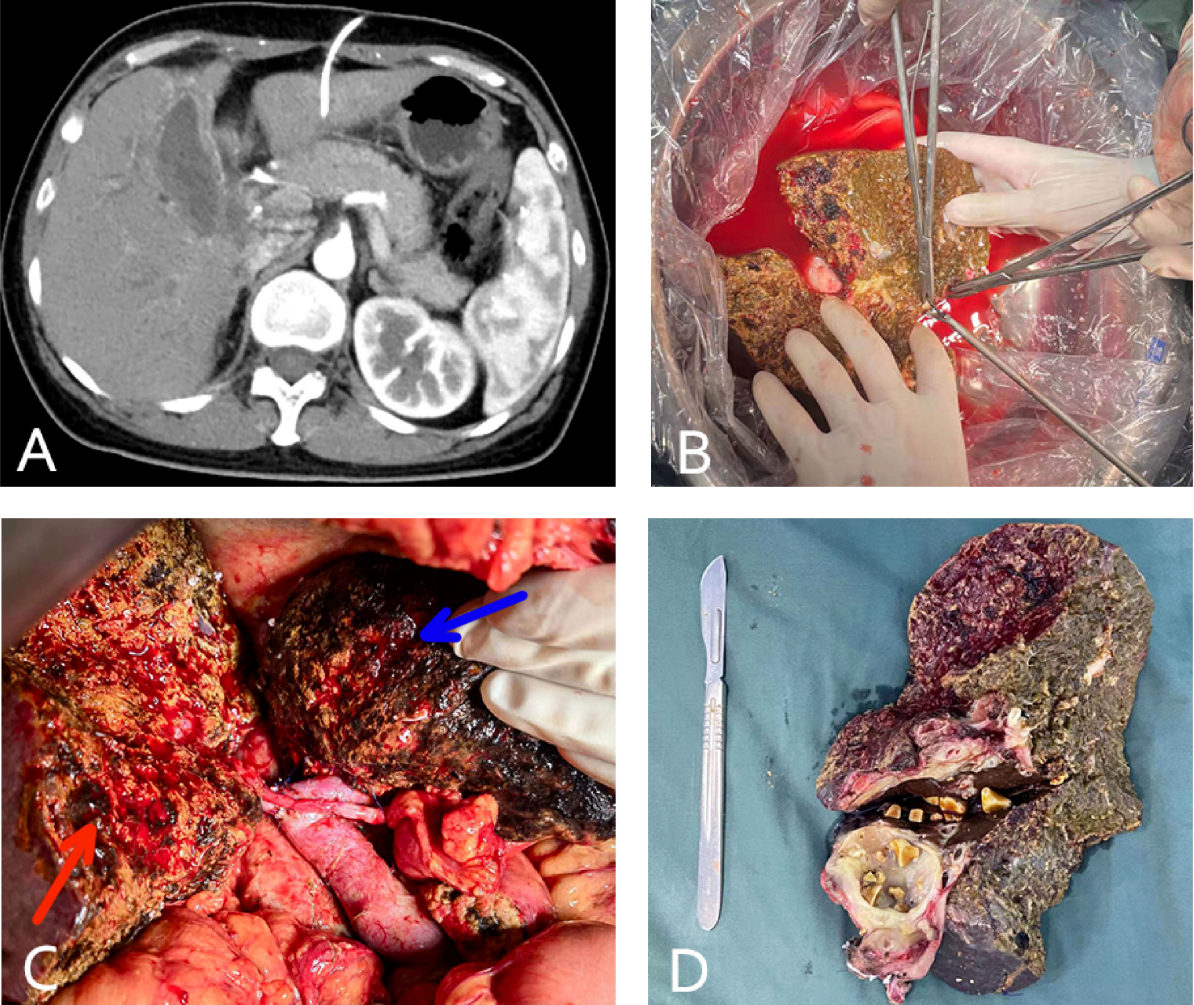
(case 7). A 46-year-old female gallbladder carcinoma patient with invasion of the hilar bile duct, antrum, and duodenum, and vascular involvement of the portal and right hepatic veins **(A)**. The patient underwent *in vivo* right hemihepatectomy, the tumor was resected at the back table, and an autograft of the right posterior lobe was created **(B)**. Whipple surgery and anastomosis of the portal vein and hepatic artery of the right posterior lobe (the blue arrow indicates the unresected left liver and the red arrow indicates the autograft of the posterior lobe liver) **(C)**. The excised specimen **(D)**.

Five patients (cases 1–5) with an invaded inferior vena cava received ITH-ELRAT (**Figures 1**, **2**). A schematic diagram of the surgical procedures is shown in **Figure 3**. The other five patients (cases 6–10) who received IPH-ELRAT all had BTC with invaded hilar vessels. Following multidisciplinary team discussions, conventional hepatectomy was excluded due to the high risk of a prolonged vascular blockade, severe ischemia-reperfusion injury, and bleeding. This was not enough to preserve the left liver and could have led to serious postoperative small-for-size syndrome. Portal vein embolization with associated liver partition and portal vein ligation for staged hepatectomy were also considered. However, the techniques would have wasted at least one portion of the liver and probably increased the risk of complications. Moreover, these procedures might affect preoperative systemic therapy and increase the risk of disease progression. Thus, based on our experience with liver transplantation, IPH-ELRAT (autotransplantation of the right posterior lobe of the liver) was conducted on the five patients using three major procedures (**Figure 4**). First, a right hemihepatectomy was performed. The right liver was immediately placed in a back-table sterile ice bath and HTK solution at 0–4°C was infused through the portal vein. Second, the malignant lesions were completely removed *ex vivo*. An autograft with segments VI and VII was prepared. If the remaining portal vein or hepatic artery was not sufficiently long, reconstruction was performed using artificial blood vessels or an autogenous great saphenous vein. Third, the graft was autotransplanted back into the right liver fossa. The right hepatic vein was anastomosed to the retrohepatic inferior vena cava using the end-to-side pattern and the right portal vein and hepatic artery were anastomosed to the relevant vessels using the end-to-end style. End-to-end anastomosis of the bile duct or cholangiojejunostomy was performed based on the size of the bile duct. As a result, the graft of the right posterior segment formed a partial autologous graft. The left liver remained native and was kept free of ischemia-reperfusion injury. Synchronous Whipple surgery was performed for two patients with involvement of the lower part of the common bile duct, antrum, and duodenum.

### Postoperative outcomes

The median postoperative hospital stay was 18 days (range:13–30 days). All patients received low molecular weight heparin postoperatively. If a vascular graft was used, bridging with rivaroxaban was required after discharge. If a vascular graft was not used, anticoagulant therapy was unnecessary after hospital discharge. One patient (case 1) developed right hepatic vein thrombosis four months after surgery and recovered after stent implantation using interventional therapy. Another patient (case 6) experienced pancreatic and chylous leakage. No complications related to bleeding, infection, or liver failure were observed in the perioperative period. Small-for-size syndrome did not occur in any of the patients.

### Postoperative adjuvant treatment and survival

Nine of the ten patients (90%) received postoperative adjuvant therapy. The survival rate one month after surgery was 100%. The patient (case 6) without pre- or post-operative systemic therapy died two months after surgery due to sepsis. The other nine patients (90%) are currently alive with a median follow-up of 8.5 months (range 6–16.5 months). To date, seven of the nine patients (77.78%) have had no cancer recurrence. However, the patient (case 3) with metastases from colonic carcinoma experienced distant metastases in the backbone. Another patient (case 2) with BTC experienced liver tumor recurrence (1.4 cm) three months after surgery and was treated using microwave ablation therapy.

## Discussion

The liver cannot tolerate a long period of ischemia *in vivo*. When a tumor invades the important blood vessels of the liver, including the confluence of the hepatic veins or the inferior vena cava, conventional surgery cannot be performed (17). ELRAT can be used for a variety of benign and malignant liver lesions and is often regarded as the last treatment option (25). This treatment can effectively reduce warm ischemic injuries and allow the tumor can be removed from a bloodless area outside the body following perfusion. ELRAT has technical requirements that require multidisciplinary cooperation. The surgical team must be experienced in both the liver transplantation process and complex perioperative anesthesia management (23).

ELRAT is most often used to treat hepatic alveolar echinococcosis (19) and also suitable for large liver tumors and/or difficult anatomical locations, including severe compression or infiltration of large blood vessels or biliary structures such as the inferior vena cava, portal vein, and hepatic vein (26, 27). ELRAT is also a reasonable choice when the risk of *in situ* resection is high, due to uncontrollable massive bleeding or liver failure, and there is a need to extend the operation time (17). However, ELRAT has several contraindications, including cirrhosis, cholestasis, Budd-Chiari syndrome, and deficiency of residual liver volume after lesion resection (<40%) (28). Relative contraindications include two conditions: 1. hepatic echinococcosis with extrahepatic lesions that can be controlled by albendazole; 2. hepatic malignancy with limited extrahepatic metastasis that can be controlled using systemic therapy (such as chemotherapy, targeted therapy, and immunotherapy) (29).

The most appropriate resection option should be carefully planned prior to surgery to reduce or avoid complications. Residual liver volume should be evaluated quantitatively and qualitatively before surgery to avoid small liver syndrome (30). The present study reported the first five cases of patients with malignant tumors who received IPH-ELRAT. The advantages of this procedure are its ability to preserve a high amount of liver parenchyma to avoid small-for-size syndrome and the lack of a need for postoperative immunosuppressants or organ donors (20). Compared to autologous liver transplantation after total hepatectomy, autologous liver transplantation after partial hepatectomy often involves more delicate and complex vascular and bile duct reconstruction. In our study, two patients who underwent autologous liver transplantation after partial hepatectomy underwent synchronous Whipple procedure, which resulted in longer operation time and greater amount of blood loss. However, compared with total hepatectomy, patients with partial hepatectomy had more stable intraoperative hemodynamics due to the retention of the left liver during *ex vivo* tumor resection, as well as the no need for resection and reconstruction of the inferior vena cava. The procedure of partial hepatectomy *in vivo* also avoids cold ischemia-reperfusion injury for the retention of the left liver.

While ELRAT has had increasing success as a treatment for patients with benign and malignant liver tumors, the risk of serious complications and even death during the perioperative period remains high. Previous studies have shown that the prognosis of patients with liver malignancies after ELRAT remains disappointing due to the high rates of recurrence and mortality after surgery (19). To date, the use of ELRAT for BTC patients remains limited (15, 26, 29, 31). Ameta-analysis of 53 studies included 244 patients who underwent ELRAT, of whom 160 (65.6%) had benign tumors and 84 (34.4%) had malignancies. The most common malignant lesions were colorectal cancer metastases (11.5%) and cholangiocellular carcinomas (9.4%) (19). The 30-day and 90-day mortality rate in the malignant subgroup was 11.3% and 21.6%, respectively, the one-year survival rate was 65.0%, and the rate of tumor recurrence during follow-up was 34.4% (19). Of the 23 cholangiocellular carcinoma cases, 21 had invasion of the major vessels. Four patients with intrahepatic cholangiocarcinomas (17.4%) died within 30 days of surgery, and only eight survived 12 months after surgery. Three patients experienced tumor recurrence. Liver allotransplantation was performed in eight (34.8%) patients (19). Another recent study demonstrated relatively favorable outcomes (15). The current study reported on ten patients who received ELRAT for conventionally unresectable hepatobiliary primary cancers or hepatic metastases, including eight with BTC. Encouragingly, the survival rate one month after surgery was 100%. One patient with BTC died from sepsis after two months while the remaining patients are currently alive, with a median follow-up of 8.5 months (range 6–16.5 months). To date, seven of the nine patients (77.78%) have had no cancer recurrence, including six with BTC.

In recent years, the efficacy of systematic treatments, including PD-1 inhibitors, targeted therapy, and chemotherapy, has been confirmed by many studies of hepatobiliary cancers and liver metastases of gastrointestinal malignant tumors, allowing large and initially unrectable tumors to become resectable (7, 11). Six of the ten patients in this study received preoperative systematic therapy, which effectively controlled the tumor and made radical surgery possible. This study also reports on the first four patients with BTC who were treated with systematic therapy using a combination of PD-1 inhibitor, lenvatinib, and chemotherapy, and then successfully received ELRAT with good outcomes.

## Conclusion

ELRAT is a complex surgery that can facilitate radical resection in patients with malignancies. However, the effectiveness of this surgery remains controversial. In recent years, progress in the development of systematic therapies, such as PD-1 inhibitors and targeted treatment, may promote the use of ELRAT. The current study demonstrated relatively favorable outcomes and reported on the first five patients to receive IPH-ELRAT for advanced malignancies. These findings suggest that this may provide a new surgical option for patients with conventionally unresectable hepatobiliary malignancies, while also helping to alleviate organ shortages.

## Data availability statement

The original contributions presented in the study are included in the article/supplementary material. Further inquiries can be directed to the corresponding author.

## Ethics statement

The studies involving human participants were reviewed and approved by the Institutional Review Board of the Second Affiliated Hospital, School of Medicine, Zhejiang University. The patients/participants provided their written informed consent to participate in this study. Written informed consent was obtained from the individual(s) for the publication of any potentially identifiable images or data included in this article.

## Author contributions

SX, CH, and ZJ collected case data. SX and CH wrote the manuscript. GL, BZ, ZG, WW and SY proofread and revised the manuscript. All authors contributed to the article and approved the submitted version.

## Glossary

**Table:**

ELRAT	ex vivo liver resection and autotransplantation
ITH-ELRAT	*in vivo* total hepatectomy and then ex vivo liver resection and autotransplantation
IPH-ELRAT	*in vivo* partial hepatectomy and then ex vivo liver resection and autotransplantation
BTC	biliary tract cancer
PD-1	anti-programmed cell death protein 1
HCC	hepatocellular carcinoma
ORR	objective response rate
CRC	colorectal carcinoma
GIST	gastrointestinal stromal tumor
HTK	histidine-tryptophan-ketoglutarate
PV	portal vein
LPV	left portal vein
RPV	right portal vein
LHA	left hepatic artery
RHA	right hepatic artery
LHV	left hepatic vein
RHV	right hepatic vein
MHV	middle hepatic vein
IVC	inferior vena cava.
