# Corrigendum: *In vivo* total or partial hepatectomy followed by *ex vivo* liver resection and autotransplantation for malignant tumors: a single center experience

**DOI:** 10.3389/fonc.2023.1251636

**Published:** 2023-08-11

**Authors:** Shaoyan Xu, Chenlu Hu, Zedong Jiang, Guogang Li, Bo Zhou, Zhenzhen Gao, Weilin Wang, Sheng Yan

**Affiliations:** ^1^ Division of Hepatobiliary and Pancreatic Surgery, Department of Surgery, Second Affiliated Hospital, School of Medicine, Zhejiang University, Hangzhou, Zhejiang, China; ^2^ Key Laboratory of Precision Diagnosis and Treatment for Hepatobiliary and Pancreatic Tumor of Zhejiang Province, Second Affiliated Hospital of Zhejiang University School of Medicine, Hangzhou, Zhejiang, China; ^3^ Department of Nursing, Second Affiliated Hospital, School of Medicine, Zhejiang University, Hangzhou, Zhejiang, China

**Keywords:** *in vivo* total hepatectomy, *in vivo* partial hepatectomy, hepatic metastases, autotransplantation, biliary tract cancer

**Error in Author List**

In the published article, there was an error in the author list, and author Weilin Wang was erroneously excluded. The corrected author list appears below.

Shaoyan Xu ^1,2†^, Chenlu Hu ^3†^, Zedong Jiang ^1,2^, Guogang Li ^1,2^, Bo Zhou^1,2^, Zhenzhen Gao ^1,2^, Weilin Wang^1,2*^ and Sheng Yan ^1,2*^

The authors apologize for this error and state that this does not change the scientific conclusions of the article in any way. The original article has been updated.

**Text Correction**

In the published article, there was an error in the Correspondence section where one corresponding author was erroneously excluded. In addition to author Sheng Yan, also Weilin Wang is a corresponding author.

The correct corresponding authors are the below:

Sheng Yan, shengyan@zju.edu.cn

Weilin Wang (wam@zju.edu.cn)

The authors apologize for this error and state that this does not change the scientific conclusions of the article in any way. The original article has been updated.

**Text Correction**

In the published article, there was an error in the Author Contributions where author Weilin Wang was erroneously excluded.

The Author Contributions previously stated:

“SX, CH, and ZJ collected case data. SX and CH wrote the manuscript. GL, BZ, ZG, and SY proofread and revised the manuscript. All authors contributed to the article and approved the submitted version.”

The corrected Author Contributions appears below.

**Author Contributions**

SX, CH, and ZJ collected case data. SX and CH wrote the manuscript. GL, BZ, ZG, WW and SY proofread and revised the manuscript. All authors contributed to the article and approved the submitted version.

The authors apologize for this error and state that this does not change the scientific conclusions of the article in any way. The original article has been updated.

